# Dissecting *Solidago canadensis*–soil feedback in its real invasion

**DOI:** 10.1002/ece3.2743

**Published:** 2017-03-09

**Authors:** Li‐Jia Dong, Jian‐Xia Yang, Hong‐Wei Yu, Wei‐Ming He

**Affiliations:** ^1^ State Key Laboratory of Vegetation and Environmental ChangeInstitute of BotanyChinese Academy of SciencesBeijingChina; ^2^ College of Resources and EnvironmentUniversity of Chinese Academy of SciencesBeijingChina

**Keywords:** abiotic effect, biotic effects, community structure and function, competitive tolerance and suppression, invader–soil feedback, structural equation modeling

## Abstract

The importance of plant–soil feedback (PSF) has long been recognized, but the current knowledge on PSF patterns and the related mechanisms mainly stems from laboratory experiments. We aimed at addressing PSF effects on community performance and their determinants using an invasive forb *Solidago canadensis*. To do so, we surveyed 81 pairs of invaded versus uninvaded plots, collected soil samples from these pairwise plots, and performed an experiment with microcosm plant communities. The magnitudes of conditioning soil abiotic properties and soil biotic properties by *S. canadensis* were similar, but the direction was opposite; altered abiotic and biotic properties influenced the production of subsequent *S. canadensis* communities and its abundance similarly. These processes shaped neutral *S. canadensis*–soil feedback effects at the community level. Additionally, the relative dominance of *S. canadensis* increased with its ability of competitive suppression in the absence and presence of *S. canadensis*–soil feedbacks, and *S. canadensis*‐induced decreases in native plant species did not alter soil properties directly. These findings provide a basis for understanding PSF effects and the related mechanisms in the field conditions and also highlight the importance of considering PSFs holistically.

## 1 Introduction

The process in which plants can alter the properties of their surrounding soils and these changes in turn influence the performance of the same or other plants is viewed as a plant–soil feedback (PSF) (Bever, Westover, & Antonovics, 1997; Ehrenfeld, Ravit, & Elgersma, 2005). PSF has now become an important concept when trying to understand plant population dynamics, plant community properties, and functioning of terrestrial ecosystems (Bailey & Schweitzer, 2016; van Nuland et al., 2016; van der Putten, Bradford, Brinkman, van de Voorde, & Veen, 2016; van der Putten et al., 2013), and received increasing attention (Bailey & Schweitzer, 2016; Kulmatiski, Beard, Stevens, & Cobbold, 2008; Levine, Pachepsky, Kendall, Yelenik, & HilleRisLambers, 2006; Meisner et al., 2014; van Nuland et al., 2016; Suding et al., 2013). Positive PSF can result from enhanced nutrient availability (Levine et al., 2006) or the accumulation of symbiotic mutualists (Callaway, Thelen, Rodriguez, & Holben, 2004; Klironomos, 2002), working as a homogenizing force. For example, positive PSF can help invasive plants to dominate over native plants (Kulmatiski et al., 2008; Levine et al., 2006; Meisner et al., 2014). Negative PSF can be due to nutrient immobilization or depletion (Levine et al., 2006) or the accumulation of root herbivores and soil pathogens (van der Putten, Vandijk, & Peters, 1993; Suding et al., 2013), acting as a diversifying force. For instance, negative PSF favors species coexistence (van der Putten et al., 2013). Additionally, PSF effects can also be neutral in some cases (Meisner et al., 2014; Perkins & Nowak, 2013). Relative to negative and positive PSFs, less is known about the determinants underlying neutral PSFs.

However, it should be noted that most evidence for PSFs comes from greenhouse studies or potted plants and that previous studies have focused on the responses of a single species to PSFs (Heinze, Sitte, Schindhelm, Wright, & Joshi, 2016; Meisner et al., 2014). A recent research shows that there are differences in PSFs between the greenhouse and field conditions (Heinze et al., 2016), suggesting that the PSF findings from the laboratory experiments cannot be extrapolated to the field conditions. To date, several aspects of PSFs remain poorly understood, thereby foiling our understanding of PSFs in nature. For example, do trainer species alter soil abiotic and biotic properties through direct or indirect pathways? Do altered soil abiotic and biotic properties influence the subsequent plant communities equally or disproportionally? Are PSF effects on community performance positive, negative, or neutral? Addressing these questions in the context of real situations is important for understanding population dynamics, community succession, and plant invasions. In other words, it is crucial to explore PSF patterns in the field conditions and to elucidate the associated mechanisms (Bailey & Schweitzer, 2016; van Nuland et al., 2016; van der Putten et al., 2013, 2016).

Plant invasion provides a stage for addressing these questions for a few reasons. Invasive plants can rapidly alter recipient communities so that PSFs are evident and contribute to invasion success (Klironomos, 2002; Levine et al., 2006; Suding et al., 2013). These situations enable pairwise comparisons to be feasible. Second, plant invasion tends to decrease native plant species diversity (Dong, Yu, & He, 2015; and references therein), and this process may play a key role in conditioning soils. However, this aspect has been overlooked. Finally, the subsequent communities of plant invaders can experience the soils conditioned by conspecifics or heterospecifics, and little is known about the community‐level consequences of PSFs. Here, we selected *Solidago canadensis* L. (hereafter *Solidago*) as a focal invader species for the following reasons. First, *Solidago* is one of the most noxious invasive forbs across the world so that its feedbacks with soils may play a key role in its successful invasion (Sun & He, 2010; Weber, 2003). Second, this species is among the most serious plant invaders in China, thereby resulting in huge losses economically and environmentally (Dong, Lu, Zhang, Chen, & Li, 2006). Finally, *Solidago*, as a model species, has been studied extensively (Abhilasha, Quintana, Vivanco, & Joshi, 2008; Dong, Sun, Gao, & He, 2015; Dong et al., 2006; Sun & He, 2010).

The purpose of this study was to identify PSF patterns and to explore the underlying mechanisms in a real invasion. To do so, we performed field investigations, determined a suite of soil abiotic and biotic properties, and conducted a bioassay experiment with field soils and microcosm communities consisting of *Solidago* and three Chinese natives. Here, we put forward several hypotheses. In the field conditions, long‐term *Solidago*–soil feedback may be positive for the performance of subsequent *Solidago* communities, because this effect can facilitate *Solidago* to dominate over native plant species. Altered soil abiotic and biotic properties contribute differentially to the structure and function of subsequent *Solidago* communities. This relative importance might help us to understand the role of PSFs holistically but has been overlooked in the past decade. *Solidago* invasion alters soil properties directly and indirectly, and the importance of these two pathways differs. Dissecting PSF pathways is required for elucidating its mechanisms.

## 2 Materials and methods

### 2.1 Site description and field investigation

**Figure 1:**
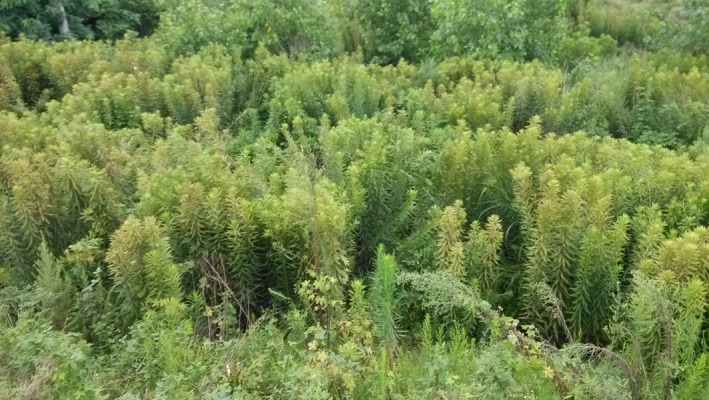
An image of a plant community invaded by *Solidago canadensis* heavily in southern China. Photograph credit: L.J. Dong

*Solidago* is a perennial forb and native to North America (Werner, Bradbury, & Gross, 1980). It produces seeds and rhizomes at the same time, and can dominate over other plants or even shape monocultures in some habitats (Werner et al., 1980). *Solidago* was introduced to China in 1935 (Dong et al., 2006) and has invaded large areas of southern China and become an overwhelming dominant in some habitats (Figure 1). In summer 2014, we selected nine sampling locations where *Solidago* invaded heavily (i.e., the cover of *Solidago* was 83.2% ± 1.4% and its plant height was 2.0 ± 0.1 m). The mean temperature and precipitation per sampling location are presented in Table S1. We selected three sites per location and surveyed three pairs of invaded and uninvaded 1 × 1 m plots per sampling site. Pairwise invaded and uninvaded plots were chosen according to the criteria proposed by Powell, Chase, and Knight (2013), and this pairwise approach has been extensively used in the related studies (Gaertner, Breeyen, Hui, & Richardson, 2009). During the investigation, we recorded all plant species and their cover, density, and height per plot.

### 2.2 Soil collection and analyses

We collected soil samples from 81 pairs of uninvaded versus invaded plots in southern China to allow them to represent diverse soil regimes. Field soil is fit for addressing PSF in natural conditions, and has been widely used in previous studies (Brinkman, Van der Putten, Bakker, & Verhoeven, 2010; Heinze et al., 2016; van der Putten et al., 1993; Rutten, Prati, Hemp, & Fischer, 2016). In each plot, five soil samples were taken from the rhizospheres of *Solidago* (i.e., invaded plots) or from the top 10 cm of the soil profile in native plant communities (i.e., uninvaded plots), and then composited as a soil sample. Each soil sample was further divided into two portions: one for determining the following abiotic properties and soil microbes, and the other for a bioassay experiment.

For soil abiotic properties, pH was determined in a soil solution of 1:2.5 (soil:distilled water) using a pH meter (Sartorius PB‐10 meter); organic carbon (OC) was determined using the potassium dichromate oxidation method; total nitrogen (TN) was determined using the Kjeldahl apparatus (FOSS 2200); available phosphorus (AP) was determined using a UV‐2550 ultraviolet spectrophotometer; and ammonia (NH_4_‐N) and nitrate (NO_3_‐N) were determined using a continuous flow analyzer (Dong, Sun, et al., 2015). Soil texture was determined using a laser particle size analyzer (Mastersizer, 2000).

For soil microbes, we employed phospholipid fatty acid (PLFA) analysis (Dong, Yu, et al., 2015). The fatty acids chosen to represent fungi were 18:2ω6,9c and 16:1ω5c, to represent bacteria were i14:0, 14:0, i15:0, a15:0, 15:0, a16:0, i16:0, 16:0, 16:1ω7c, 16:1ω9c, i17:0, a17:0, 17:0, cyl7:0, 18:0, 18:1ω5c, 18:1ω7c, and cyl9:0, and to represent actinomyces were 10Me16:0, 10Me18:0, and 10Me20:0. The ratio of fungi to bacteria was calculated (Bossio & Scow, 1998; Larsen & Bodker, 2001).

### 2.3 Soil bioassay: Microcosm community experiment

We conducted an experiment with microcosm plant communities consisting of *Solidago* and/or Chinese native plant species. The native plants were: *Cichorium intybus* (Asteraceae, perennial forb), *Poa pratensis* (Poaceae, perennial grass), and *Setaria plicata* (Poaceae, annual grass). These natives were chosen in this experiment as they commonly occur in southern China and also appear in plant communities invaded by *Solidago* in China. In the experiment, *Solidago* was grown at the center of cylindrical pots either alone or with these natives (one *Solidago* individual and three native individuals per pot, forming a microcosm plant community), and the three natives were also grown in pots as controls. Each species combination was subjected to each of the four treatments: (1) regular uninvaded soils from native plant communities (i.e., uninvaded plots), (2) regular invaded soils from *Solidago* communities (i.e., invaded plots), (3) uninvaded soils sterilized with a dose of 40 kGy of gamma radiation, and (4) invaded soils sterilized with a dose of 40 kGy of gamma radiation. All plants from seed were grown in 250‐mL pots. As our goal was to contrast the performance of microcosm plant communities, we repeated the four soil treatments through using different soil samples from plots. Accordingly, there were 81 pots per treatment in this experiment. All the pots were put in a greenhouse at the Institute of Botany, Chinese Academy of Sciences, where temperatures and humidity were maintained between 20–30°C and 50%–60%, and photosynthetically active radiation during the day remained above 1200 μmol m^−2^ s^−1^. During the experiment, water was supplied to all plants as required, and the other growing conditions were identical for all plants. Note that no soil leaching occurred and no nutrients were supplied during the experiment. This experiment lasted for five months. At the end of the experiment, all plants were harvested, separated into shoots and roots, rinsed, oven‐dried at 85°C for 48 h, and weighed. The total dry biomass of a plant was equal to the sum of dry root biomass and dry shoot biomass.

### 2.4 Data analyses

We mainly focused on the performance of microcosm plant communities consisting of *Solidago* and three Chinese natives so that we termed this community as a *Solidago* community below. The total biomass of a community equaled the sum of the total dry biomass of each plant species in the community. We calculated the relative abundance of *Solidago* in a *Solidago* community as follows:

(1) $$\mathrm{Abundance}\;=\;\frac{\mathrm{Total}\;\mathrm{biomass}\;\mathrm{of}\;Solidago}{\mathrm{Total}\;\mathrm{biomass}\;\mathrm{of}\;Solidago\;\mathrm{community}}\times 100\%$$

We considered competitive tolerance and suppression at the same time. The former indicates the ability of a plant to avoid being suppressed by its neighbors and the latter indicates the potential of a plant to suppress its neighbors (Weigelt & Jolliffe, 2003). These two abilities coshape the competitive ability of a plant (Weigelt & Jolliffe, 2003). Accordingly, we coined a competitive tolerance index (CTI) and a competitive suppression index (CSI), respectively. The greater these two indices are, the stronger the competitive ability is. These two indices can better meet our demand and can be expressed as follows:

(2) CTI=(BSw−BSo)/(BSw+BSo)

(3) CSI=(BNo−BNw)/(BNo+BNw)

where BSw is the biomass of *Solidago* grown with natives and BSo is the biomass of *Solidago* without natives. BNo is the biomass of natives without *Solidago* and BNw is the biomass of natives grown with *Solidago*.

In the regular soil, the plant community performance was determined by both soil abiotic and biotic properties, representing the total effect of a soil (i.e., the total effect of a regular soil). In the sterilized soil, the community performance was determined by soil abiotic properties only, representing the effect of soil abiotic properties (i.e., the abiotic effect of a sterilized soil). The total effect of a soil was determined through directly measuring the traits (i.e., biomass, abundance, and competitive ability) of a microcosm community in the regular soil, and the abiotic effect of a soil was determined through directly measuring the traits of a microcosm community in the sterilized soil. According to the above definitions of the total effect and abiotic effect, we calculated the biotic effect of a soil as follows:

(4) $$\mathrm{Biotic}\mathrm{effect}\;=\;{\mathrm{GC}}_{\mathrm{total}}-{\mathrm{GC}}_{\mathrm{abiotic}}$$

where GC_*total*_ represents the performance of *Solidago* grown in a regular soil and GC_*abiotic*_ represents the performance of *Solidago* grown in a sterilized soil.

However, it should be, in particular, noted that our goal was to quantify the role of *Solidago*–soil feedbacks in the abiotic effect, biotic effect, and total effect. Consequently, we tested whether the role of *Solidago*–soil feedbacks was significant through contrasting the soil abiotic effect, soil biotic effect, and total soil effect between invaded soils and uninvaded soils.

All natives were pooled together when analyzing data. One‐way analysis of variance was used to test the effects of a soil conditioned by *Solidago* on its subsequent community biomass, relative abundance, competitive tolerance ability, and competitive suppression ability. A model II regression was used to test the relationships between the relative dominance of *Solidago* in *Solidago* communities consisting of *Solidago* and three Chinese natives and both its competitive tolerance ability and suppression ability.

To address how *Solidago* invasion altered soil properties and how these changes in turn influenced *Solidago* community production and its relative abundance, we used the partial least squares path modeling (PLS‐PM) algorithm. According to the PSF theories (Ehrenfeld et al., 2005), *Solidago* invasion can alter soil abiotic and biotic properties directly or indirectly, and altered soil abiotic and biotic properties in turn affect its subsequent community production and dominance. Based on these processes, we therefore defined the initial structural model.

The path model was developed in a formative way. We defined *Solidago* invasion, native plant species, soil abiotic properties, soil biotic properties, community production, and *Solidago* dominance as six latent variables (LVs, an abstract concept). In the initial model, we set manifest variables (MVs, measured variables) for each LV. For example, to quantify the intensity of *Solidago* invasion, we coined a relative invasion index (RII) as follows:

(5) $$\mathrm{RII}\;=\;\left\{\frac{Ci}{\mathrm{MAX}(Ci)}\;+\;\frac{Di}{\mathrm{MAX}(Di)}+\frac{Hi}{\mathrm{MAX}(Hi)}\right\}/3$$

where *Ci*,* Di*, and *Hi* represent cover, density, and height of *Solidago* in invaded plots, respectively. Therefore, *Solidago* invasion included only one component (RII) as MV. Native plant species included four components: changes in species richness, Shannon–Wiener index, Pielou evenness index, and dominance index. These diversity indices were determined as described by Magurran (1988), and their relative changes were calculated as follows:

(6) Changes=(Ti−Tu)/(Ti+Tu)

where Ti represents a diversity index in invaded soils, while *Tu* represents a diversity index in uninvaded soils. Soil abiotic properties included changes in pH, OC, TN, NH_4_‐N, NO_3_‐N, AP, and texture. Soil biotic properties included changes in fungi, bacteria, actinomyces, total PLFAs, and fungi:bacteria ratio. Both community production and *Solidago* dominance included one component: change in the total biomass and change in species abundance. The calculating procedures about the changes in soil abiotic and biotic properties, community production, and *Solidago* dominance were the same as native plant species described above. Here, each LV was considered as a linear combination of its own MVs (Tenenhaus, Esposito Vinzi, Chatelin, & Lauro, 2005).

After the initial model including all possible MVs (indicators) was fitted, we simplified the model through assessing the indicator reliability to maximize the efficiency of PLS‐PM (Chin & Dibbern, 2010). Indicator reliability is usually determined by the construct loadings, and we selected those MVs with their loadings significant at the 0.01 level and above the recommended 0.7 parameter value for each LV. Here, the loadings greater than 0.7 (i.e., communality values greater than 0.7^2^ = 0.49) are considered as acceptable, because “communalities represent the amount of variability explained by a latent variable and a communality greater than 0.5 means that more than 50% of the variability in an indicator is captured by its latent construct” (Tenenhaus et al., 2005; Chin & Dibbern, 2010). Model parameters (the path coefficients [β], the loadings, and communalities of the MVs) and fit indices (*R* ^2^) were validated by bootstrapping. The significance of the path coefficients was estimated at the 0.1 level (Chin & Dibbern, 2010).

All statistical analyses were carried out using R 3.3.0. A Model II regression was performed using the package “smatr”. PLS‐PM algorithm was performed using the package “plspm”.

## 3 Results

### 3.1 *Solidago*–soil feedback effects

**Figure 2:**
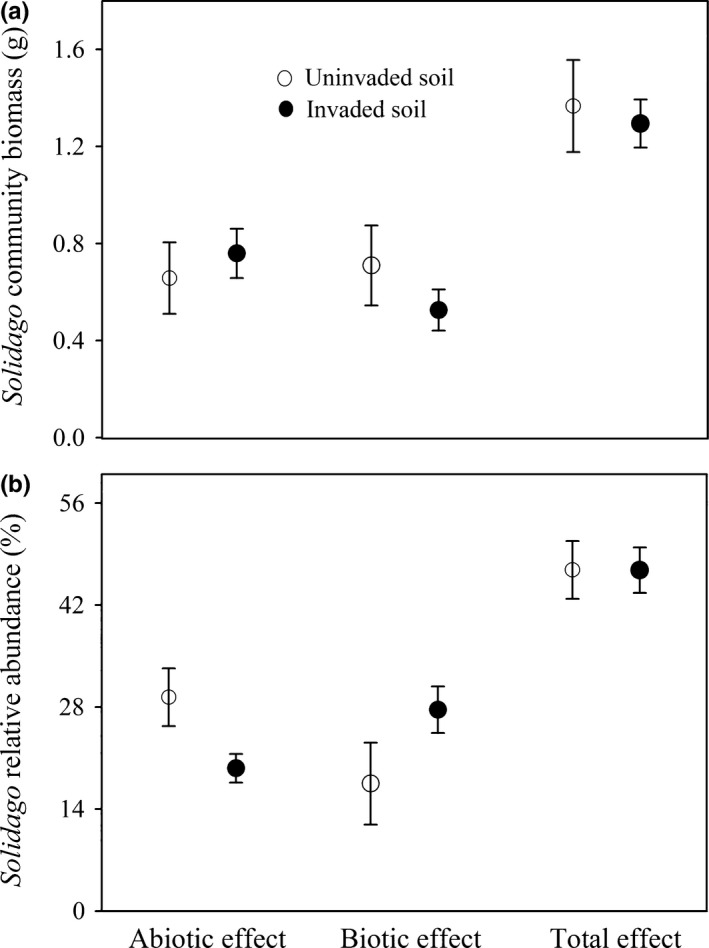
Total biomass of microcosm *Solidago* communities (also termed as mixtures: plant communities consisting of *Solidago* and three Chinese natives) (a) and relative abundance of *Solidago* in mixtures (b) grown in uninvaded and invaded soils. Data are means ± 1 SE (*n* = 81). Abiotic effect, biotic effect, and total effect in two soils indicate how *Solidago*‐induced changes in soil abiotic properties, soil biotic properties, and soil abiotic and biotic properties influence the *Solidago* community production and the relative abundance of *Solidago* in mixtures. See Section 2 for more details on determining abiotic effect, biotic effect, and total effect

Soil nutrients and microbes were variable depending on sampling locations (Tables S2 and S3). In terms of the total biomass of *Solidago* communities consisting of *Solidago* and three Chinese natives, the abiotic effect, biotic effect, and total effect were similar between invaded soils and uninvaded soils (Table 1: all *p *>* *.05; Figure 2a). Accordingly, *Solidago*–soil feedbacks had no influences on its subsequent community production. The relative abundance of *Solidago* was smaller in invaded soils than in uninvaded soils when soil abiotic properties were present only, and the opposite was the case when soil biotic properties were present only (Table 1: both *p *<* *.05; Figure 2b). The relative abundance of *Solidago* was similar between invaded regular soils and uninvaded regular soils (Table 1: *p *=* *.994; Figure 2b), suggesting that soil abiotic effects and soil biotic effects could offset each other.

**Table 1:** One‐way analysis of variance of *Solidago* community biomass, *Solidago* relative abundance, and the competitive tolerance ability and competitive suppression ability of *Solidago* in uninvaded soils (i.e., control) and invaded soils (i.e., *Solidago*–soil feedback). Abiotic effect, biotic effect, and total effect refer to the effects on *Solidago* communities of the presence of (1) soil abiotic properties, (2) soil biotic properties, and (3) soil abiotic and biotic properties. *Solidago*–soil feedback was treated as a fixed factor when analyzing data

	*df*	*F*	*p*	Residuals
*Solidago* community biomass				
Abiotic effect	1	0.272	.603	0.775
Biotic effect	1	1.101	.296	0.622
Total effect	1	0.124	.726	0.839
*Solidago* relative abundance				
Abiotic effect	1	5.667	**.019**	340.060
Biotic effect	1	3.904	**.037**	434.544
Total effect	1	0.000	.994	702.002
Competitive tolerance ability				
Abiotic effect	1	0.001	.971	0.138
Biotic effect	1	1.363	.246	0.222
Total effect	1	5.120	**.026**	0.103
Competitive suppression ability				
Abiotic effect	1	6.834	**.010**	0.177
Biotic effect	1	6.944	**.010**	0.271
Total effect	1	1.314	.254	0.112

Residuals of the table represent the residuals of mean square. Values of P < 0.05 are in bold.

**Figure 3:**
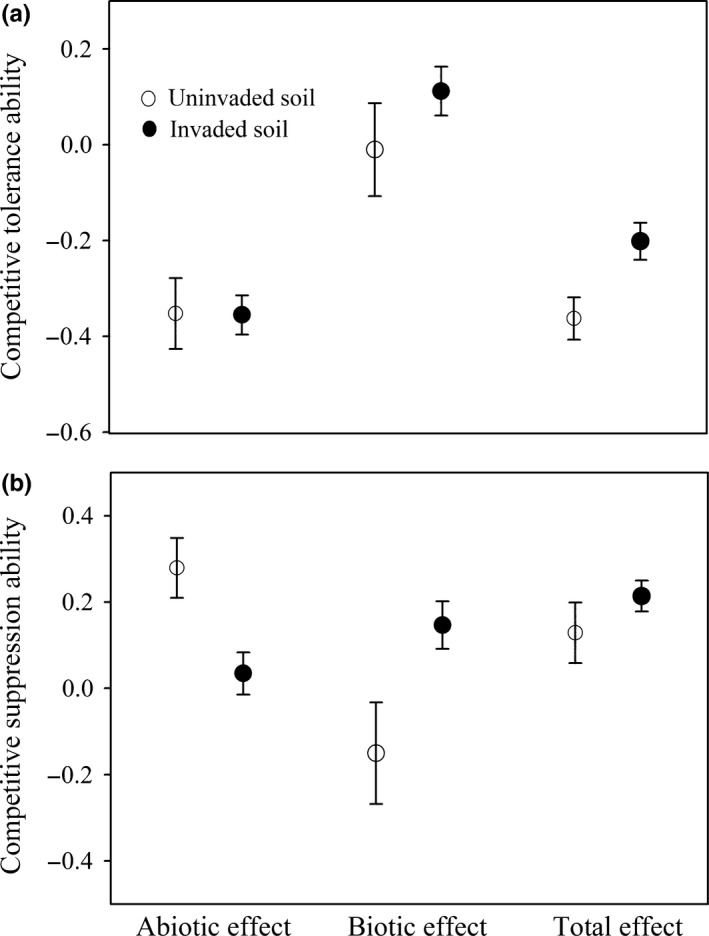
Competitive tolerance ability of *Solidago* to native plants in mixtures (i.e., plant communities consisting of *Solidago* and three Chinese natives) (a) and competitive suppression ability of *Solidago* against native plants in mixtures (b) grown in uninvaded and invaded soils. Data are means ± 1 SE (*n* = 81). Abiotic effect, biotic effect, and total effect in two soils indicate how *Solidago*‐induced changes in soil abiotic properties, soil biotic properties, and soil abiotic and biotic properties influence the competitive tolerance and suppression ability of *Solidago* in mixture*s*. See Section 2 for details on determining abiotic effect, biotic effect, and total effect

In terms of the competitive tolerance ability of *Solidago*, the soil abiotic effect and soil biotic effect did not vary with soil sources (Table 1: both *p *>* *.05; Figure 3a); however, this tolerance ability was greater in invaded regular soils than in uninvaded regular soils (Table 1: *p *=* *.026; Figure 3a). Like the relative abundance of *Solidago*, the effects of soil abiotic and biotic properties on its competitive suppression ability varied with soil sources (Table 1: both *p *<* *.05; Figure 3b), but the total effect of soil abiotic and biotic properties as a whole did not vary with soil sources (Table 1: *p *=* *.254; Figure 3b).

There were no significant correlations between the relative abundance of *Solidago* and its competitive tolerance ability in both uninvaded and invaded sterilized soils (Table 2: both *p *>* *.05). In contrast, there were significant correlations between the relative abundance of *Solidago* and its competitive tolerance ability in both uninvaded and invaded regular soils (Table 2: *p *=* *.046, *p *<* *.001); however, the slopes were similar between uninvaded and invaded regular soils (*p *=* *.182). There were no significant correlations between the relative abundance of *Solidago* and its competitive suppression ability in uninvaded sterilized soils (Table 2: *p *=* *.475) but significant in invaded sterilized soils (Table 2: *p *=* *.020). There were significant correlations between the relative abundance of *Solidago* and its competitive suppression ability in both uninvaded and invaded regular soils (Table 2: both *p *<* *.001); however, there was no significant difference in slopes between uninvaded and invaded regular soils (*p *=* *.201).

**Table 2:** Model II regression analysis of the relationships between the relative abundance of *Solidago* in mixtures (i.e., plant communities consisting of *Solidago* and three Chinese natives) and both its competitive tolerance ability and its competitive suppression ability. Control, regular soils; sterilization, sterilized soils. Values of P < 0.05 are in bold

	*Solidago* relative abundance ~ Competitive tolerance ability			
	Intercept	Slope	*R* ^2^	*p*
Invaded soil				
Sterilization	34.77	42.55	0.017	.254
Control	67.91	67.33	0.32	**<.001**
Uninvaded soil				
Sterilization	10.47	−53.58	0.015	.537
Control	79.24	89.35	0.15	**.046**

	*Solidago* relative abundance ~ Competitive suppression ability			
	Intercept	Slope	*R* ^2^	*p*
Invaded soil				
Sterilization	18.09	34.56	0.070	**.020**
Control	35.50	70.00	0.28	**<.001**
Uninvaded soil				
Sterilization	45.33	−57.28	0.021	.475
Control	39.60	56.26	0.56	**<.001**

### 3.2 Pathways of *Solidago*–soil feedback

**Figure 4:**
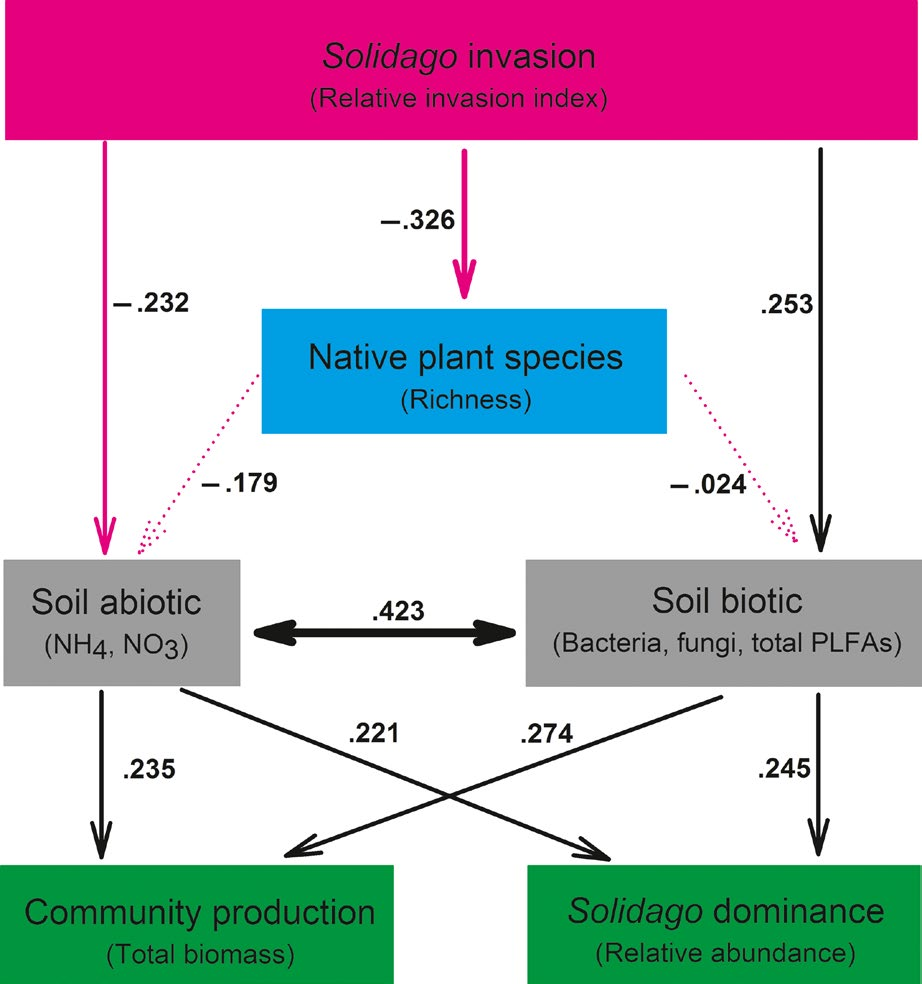
Path models examining how *Solidago* invasion alters soil abiotic properties and soil biotic properties through direct and indirect pathways and how these changes in soil abiotic and biotic properties in turn influence the production of subsequent *Solidago* communities (i.e., mixtures: plant communities consisting of *Solidago* and three Chinese natives) and the relative abundance of *Solidago* in mixtures. Solid and dashed arrows indicate significant and no significant relationships between latent variables at the level *P *=* *.1, respectively. Red and black arrows indicate negative and positive relationships between latent variables. Numbers associated with pathways between variables represent standardized path coefficients

In the field, *Solidago* invasion significantly decreased native plant species richness (β* *= −0.326, *p *=* *.005), but this decrease did not alter soil abiotic properties and soil biotic properties (Figure 4: dashed arrows; both *p *>* *.1). *Solidago* invasion had negative influences on soil abiotic properties (β* *= −0.232, *p *=* *.065), particularly soil N availability, and positive influences on soil biotic properties (β* *= 0.253, *p *=* *.033), particularly soil microbes; however, the strengths of both influences, as indicated by path coefficients, were similar (Figure 4). There were strong interactions between soil abiotic properties and soil biotic properties (Figure 4: β* *= 0.423, *p *<* *.001). Accordingly, *Solidago* invasion could influence soil biotic properties via changing soil abiotic properties, and vice versa.

Overall, the changes in soil abiotic properties and soil biotic properties altered the production of subsequent *Solidago* communities and the relative dominance of *Solidago* directly and indirectly; these strengths were similar on the basis of path coefficients. For community production, the contribution of altered soil abiotic properties was similar to that of altered soil biotic properties (Figure 4: β* *= 0.235, *p *=* *.051; β* *= 0.274, *p *=* *.023); for the relative dominance of *Solidago*, the contribution of the former was also similar to the contribution of the latter (Figure 4: β* *= 0.221, *p *=* *.070; β* *= 0.245, *p *=* *.045). Taken together, *Solidago* invasion had opposite effects on soil abiotic and biotic properties so that the effects of altered soil biotic properties on community production and species dominance were offset by those of altered soil abiotic properties. Thus, the total effects of *Solidago* invasion on its subsequent community production and dominance were not significant from zero (0.011 and 0.008; both *p *>* *.1).

## 4 Discussion

In this study, we attempted to disentangle the abiotic and biotic effects of PSFs and to dissect their direct and indirect pathways, thereby providing evidence for community‐level PSF effects and the underlying mechanisms in a real invasion. Thus, we focused on field soils from pairwise invaded and uninvaded plots. This approach enables us to address PSFs in the field conditions because field soil is less artificial and more feasible than experimentally trained soil (Brinkman et al., 2010; Rutten et al., 2016).

Plant invaders can alter the abiotic and biotic properties of their surrounding soils and the associated functions (Dong, Sun, et al., 2015; Kourtev, Ehrenfeld, & Haggblom, 2002; Perkins & Nowak, 2013). We observed that *Solidago*–soil feedback effects were neutral for the structure and function of subsequent *Solidago* communities, not supporting our first hypothesis. Such a neutral effect has been detected in other controlled experiments (Meisner et al., 2014; Perkins & Nowak, 2013). Path analyses could help to explain this neutral PSF effect. The strengths of conditioning soil abiotic and biotic properties by *Solidago* were similar, but the direction was opposite; altered soil abiotic and biotic properties in turn influenced community production and species abundance similarly. Thus, these processes shaped a neutral feedback. Overall the production and species abundance of subsequent *Solidago* communities were not affected by the soils conditioned by *Solidago*, but the associated mechanisms differed. Specifically, abiotic and biotic effects of *Solidago*–soil feedback were not significant for community production so that the total effect was not significant yet; in contrast, abiotic and biotic effects of *Solidago*–soil feedback were significant but were opposite for the relative abundance of *Solidago*, allowing the total effect to be neutral due to mutual offset. Recently, we conducted a controlled experiment and found that the total effects of two‐year *Solidago*–soil feedbacks were negative for its subsequent growth and this negative effect was linked to decreased soil N availability and changes in soil microbes (Dong, Sun, et al., 2015). In this field study, we observed a suit of changes in soil nutrients and microbes (Tables S2 and S3); however, the net effect of these changes was neutral. The differences from different studies may provide insights into PSFs. For example, PSF effects may depend on culturing conditions (e.g., artificial or natural culturing) and the duration of PSF (e.g., short‐ or long‐term PSF). In other words, PSF effects seem to have strong context dependence. Additionally, PSF effects are variable depending on invasion stages (Diez et al., 2010; Kardol, Bezemer, & van der Putten, 2006).

Perkins and Nowak (2013) used path analysis to examine PSF mechanisms, but they did not discern the direct versus indirect pathways of PSFs. We observed that *Solidago* invasion altered soil abiotic and biotic properties mainly via direct pathways. Contrary to current thought, decreased native species due to *Solidago* invasion did not alter soil abiotic and biotic properties significantly. Consequently, such indirect pathways conditioning soil may be unimportant. Interestingly, there were strong interactions between soil abiotic and biotic properties on the basis of path coefficients between them. Our findings suggest that *Solidago* invasion can alter soil properties through direct and indirect pathways at the same time, but the importance of direct pathways appears to be greater than that of indirect pathways.

It is well documented that PSF can be mediated through soil microbes (cf. Casper et al., 2008) and soil nutrients (Dong, Sun, et al., 2015; Levine et al., 2006). Our results suggest that the changes in soil nutrients and soil microbes contribute similarly to community productivity and species abundance. To our knowledge, this study is first to quantify the relative importance of soil abiotic and biotic alterations by trainer plant species in the field. Our findings indicate that soil abiotic and biotic changes are equally important for *Solidago*–soil feedback patterns in its real invasion. Accordingly, it is needed to consider soil abiotic and biotic properties holistically when addressing PSF mechanisms and should not ascribe PSF effects to soil abiotic or biotic changes simply.

The competitive ability of a plant encompasses two aspects: suppression and tolerance (Weigelt & Jolliffe, 2003). The current paradigm that competitive suppression and tolerance have equal influences on a species’ overall competitive ability has been recently questioned (Fletcher, Callaway, & Atwater, 2016). In our study, the competitive suppression and tolerance of *Solidago* had differential influences on its competitive ability, and the underlying mechanisms were also different. For competitive tolerance ability, abiotic and biotic properties of a soil showed a synergistic effect, although abiotic effects and biotic effects were not significant; for competitive suppression ability, the positive effect of soil biotic properties was neutralized by the negative effect of soil abiotic properties. These findings suggest that *Solidago* seedlings might outcompete those seedlings of native plants in the presence of soils conditioned by *Solidago*.

Two types of relationships between the relative abundance of *Solidago* and its competitive ability were detected in our experiment. Specifically, *Solidago* abundance and its competitive suppression ability were not associated in uninvaded sterilized soils, but these two traits were correlated in invaded sterilized soils. Thus, *Solidago*–soil feedback shifted this relationship from stochastic in uninvaded sterilized soils to deterministic in invaded sterilized soils. However, in regular soils, the relative dominance of *Solidago* did not vary with soil sources (i.e., invaded versus uninvaded soils), suggesting that there are other mechanisms driving species abundance in the subsequent *Solidago* communities. For example, special traits (e.g., allelopathy) of invaders directly interfere with their neighbor species (i.e., interference competition) (Sun, Collins, Schaffner, & Muller‐Scharer, 2013). As a result, the role of resource competition and interference competition may vary with soil types.

In summary, our findings provide insights into PSF effects and their determinants in a real invasion. *Solidago* invasion altered soil abiotic and biotic properties directly and indirectly, and the direct pathways appear to be more important than indirect pathways. *Solidago*–soil feedback effects on the structure and function of its subsequent communities were neutral, which may be a novel finding. This neutral effect could be explained by the equivalent but opposite roles of altered soil abiotic and biotic properties. It is already known that PSFs can play a key role in population dynamics, community succession, and plant invasions (Diez et al., 2010; Kulmatiski et al., 2008; Levine et al., 2006; Meisner et al., 2014; van der Putten et al., 2016). There are increasing studies on *S. canadensis* (Abhilasha et al., 2008; Dong et al., 2006; Dong, Sun, et al., 2015; Sun & He, 2010; Yu, Yang, Gao, & He, 2016). Our findings add significantly to the patterns of PSFs and the associated mechanisms in a broad context. For example, the neutral PSFs might be predominant in stable communities such as heavily invaded plant communities because such an effect favors the maintenance of stability. Plant species richness may change with community succession, but this change may have limited influences on feedbacks between dominant species and their surrounding soils. The results from this study also highlight that the importance of PSFs in ecology and evolution should be considered holistically, particularly in the field conditions. Additionally, the spatiotemporal patterns of PSFs deserve increasing attention because they might help to understand the functions of ecological processes.

## Conflict of interest

None declared.
