# Supplementary material for: Dissecting Solidago canadensis–soil feedback in its real invasion
Source: Ecol Evol. 2017 Mar 9;7(7):2307–15. doi: 10.1002/ece3.2743 (PMC5383496; doi:10.1002/ece3.2743)
Supplement: Supplementary file 1 [file ECE3-7-2307-s001.docx]

**Supplementary Materials**

**Table S1** The characteristics of nine sampling locations. The mean annual precipitation and mean annual temperature of each location are presented.

| Location | Province | Altitude (m) | Latitude | Longitude | Precipitation  (mm) | Temperature  (^o^C) | Soil type | Habitat |
| --- | --- | --- | --- | --- | --- | --- | --- | --- |
| Huaian | Jiangsu | 40 | 33°37'N | 119°04'E | 1049 | 15.1 | Silt loam | River side |
| Changzhou | Jiangsu | 16 | 31°49'N | 119°56'E | 1077 | 16.5 | Silt loam | Wasteland |
| Nantong | Jiangsu | 21 | 31°49'N | 121°05'E | 1089 | 16.2 | Silt loam | Road side |
| Yingtan | Jiangxi | 53 | 28°12'N | 117°00'E | 1878 | 18.9 | Sandy loam | Road side |
| Jingdezhen | Jiangxi | 39 | 29°16'N | 117°10'E | 1695 | 18.4 | Sandy loam | Road side |
| Nanchang | Jiangxi | 44 | 28°42'N | 115°52'E | 1520 | 18.6 | Sandy loam | River side |
| Jiujiang | Jiangxi | 38 | 29°44'N | 116°01'E | 1310 | 18.1 | Loam | Road side |
| Anqing | Anhui | 19 | 30°28'N | 117°04'E | 1322 | 17.5 | Loam | Road side |
| Luan | Anhui | 73 | 31°44'N | 116°30'E | 1133 | 16.4 | Loam | Wasteland |

**Table S2** The differences of seven soil abiotic properties between invaded and uninvaded sites at the nine sampling locations (mean ± 1 SE). *F*, *P* values and residuals indicate the results of one-way analysis of variance of each soil property among nine sites.

| Location | soil pH | OC  (g/kg) | TN  (g/kg) | NH_4_  (mg/kg) | NO_3_  (mg/kg) | AP (mg/kg) | Texture |
| --- | --- | --- | --- | --- | --- | --- | --- |
| Huaian | -0.2±0.1 | -1.5±1.3 | -0.2±0.1 | 2.1±1.2 | -4.4±2.6 | -2.4±1.0 | -0.2±0.1 |
| Changzhou | 0.2±0.2 | 7.4±3.3 | 0.7±0.3 | 1.3±0.5 | 8.7±3.1 | 1.6±0.8 | -0.2±0.2 |
| Nantong | 0.1±0.03 | -6.1±5.1 | -0.4±0.3 | -0.3±0.6 | -19.1±4.9 | 0.04±0.7 | -0.2±0.1 |
| Yingtan | 0.01±0.04 | -4.8±2.8 | -0.2±0.04 | -0.5±0.1 | 0.9±0.8 | -1.3±0.6 | 0.4±0.1 |
| Jingdezhen | -0.3±0.1 | 4.5±1.9 | 0.2±0.1 | -8.4±3.2 | -1.4±0.7 | 4.2±1.3 | 0.2±0.03 |
| Nanchang | -0.05±0.04 | 0.4±0.3 | -0.02±0.02 | -0.4±0.1 | -3.0±0.3 | -1.6±0.4 | 0.1±0.04 |
| Jiujiang | -0.3±0.1 | -6.0±4.9 | -0.1±0.1 | -0.2±0.1 | -4.0±1.5 | -2.4±0.6 | -0.01±0.03 |
| Anqing | 0.2±0.1 | 2.2±2.7 | 0.1±0.1 | 3.2±3.8 | -2.4±2.7 | 1.6±0.5 | -0.03±0.03 |
| Luan | -0.3±0.1 | -0.1±1.9 | -0.03±0.05 | -1.1±0.6 | -0.8±1.3 | 1.3±0.9 | -0.1±0.03 |
| *F* | 3.200 | 2.326 | 4.021 | 3.535 | 9.007 | 7.682 | 6.306 |
| *P* | 0.004 | 0.028 | 0.001 | 0.002 | <0.001 | <0.001 | <0.001 |
| Residuals | 0.099 | 85.495 | 0.231 | 27.540 | 52.486 | 5.766 | 0.058 |

Notes: Residuals of the table represent the residuals of mean square.

**Table S3** The differences of the soil microorganism content between invaded and uninvaded soils at the nine sampling locations (mean ±1 SE). *F*, *P* values and residuals indicate the results of one-way analysis of variance of each soil property among nine sites.

| Location | Actinomyces PLFAs  (nmol g^-1^) | Fungal PLFAs  (nmol g^-1^) | Bacterial PLFAs  (nmol g^-1^) | F/B ratio | Total PLFAs  (nmol g^-1^) |
| --- | --- | --- | --- | --- | --- |
| Huaian | -0.2±0.2 | 0.3±0.3 | 3.2±1.6 | -0.003±0.010 | 4.6±2.4 |
| Changzhou | -0.1±0.5 | 0.1±0.2 | 3.4±4.5 | -0.005±0.005 | 5.6±7.1 |
| Nantong | -2.8±0.4 | -2.4±0.4 | -21.5±3.7 | -0.008±0.005 | -27.8±4.9 |
| Yingtan | 0.7±0.2 | 0.2±0.2 | 2.7±1.5 | 0.004±0.011 | 3.8±2.1 |
| Jingdezhen | -0.4±0.2 | -0.5±0.4 | -8.2±1.9 | 0.013±0.019 | -11.5±2.7 |
| Nanchang | 0.1±0.1 | 0.004±0.1 | -0.4±2.4 | 0.000±0.004 | 3.4±3.1 |
| Jiujiang | -1.4±1.1 | -0.6±0.7 | -0.3±7.4 | 0.001±0.013 | -0.3±10.3 |
| Anqing | 0.5±0.7 | 0.1±0.6 | -2.6±5.6 | 0.017±0.009 | -3.4±8.6 |
| Luan | 0.0002±0.2 | 0.3±0.2 | -1.7±3.5 | 0.020±0.007 | -2.2±5.1 |
| *F* | 4.923 | 0.978 | 5.003 | 5.524 | 3.339 |
| *P* | <0.001 | 0.460 | <0.001 | <0.001 | 0.003 |
| Residuals | 2.149 | 0.001 | 1.300 | 3.568 | 307.936 |

Notes: Residuals of the table represent the residuals of mean square.
